# Effect of BMP-6 on development and maturation of mouse preantral follicles *in vitro*

**DOI:** 10.1080/13102818.2014.996605

**Published:** 2015-01-14

**Authors:** Xiyan Wang, Li Su, Xiaoyan Pan, Jian Yao, Zhixin Li, Xuenan Wang, Bangsheng Xu

**Affiliations:** ^a^ Department of Histology and Embryology, Medical College, Nantong University, Nantong, P.R. China; ^b^ Department of Neurology, Second Clinical Hospital, Jilin University, Changchun, P.R. China; ^c^ Department of Histology and Embryology, Medical College, Jilin Medical University, Jilin, P.R. China; ^d^ Department of Reproduction, Reproductive Medicine Center of the Affiliated Hospital of Jining Medical College, Jining, P.R. China

**Keywords:** BMP-6, preantral follicles, estradiol, progesterone, steroid hormone, synthetic regulation enzyme

## Abstract

The aim of this study was to investigate the effect and mechanism of bone morphogenetic protein-6 (BMP-6) on the growth and maturation of mouse follicles *in vitro*. Preantral follicles isolated from mice were incubated with recombinant human BMP-6 (rhBMP-6) before analysis. BMP-6 expression was detected by immunofluorescence and western blot. Maturation of oocytes was observed microscopically. Estradiol (E_2_) and progesterone (P_4_) levels were measured by enzyme-linked immunosorbent assay. Expression of steroidogenesis-related genes was detected by reverse transcription quantitative polymerase chain reaction. There was a marked increase in the preantral follicles maturation in cells incubated with 50 ng/mL of rhBMP-6 for eight days, compared with the control. The levels of E_2_, P_4_ and steroidogenesis-related genes were also significantly increased in granulosa cells and theca cells cultured for 6, 10 and 11 days, respectively. Conversely, the preantral follicle maturing rate was remarkably decreased in cells incubated with 50 ng/mL of rhBMP-6 for day 11, accompanied with reduction in E_2_, P_4_ levels and steroidogenesis-related genes levels. Meanwhile, compared with the control, the maturing rate was not significantly different in cells incubated with 100 ng/mL of rhBMP-6 for day 8 or day 11. However, the E_2_ levels and its relevant regulation gene expression all increased significantly, while the P_4_ content and its relevant regulation gene expression decreased. The results indicate that BMP-6 can promote the maturation of preantral follicles *in vitro* in a concentration and time-dependent manner and may play a role in the regulation of steroid hormone synthesis and/or secretion.

## Introduction

Development and maturation of follicles are important for mouse reproduction. Follicle development and maturation are regulated by the hypothalamic–pituitary–gonadal axis, and are dependent on the interaction between oocytes, granulosa cells and theca cells. The latter cells may produce many regulatory factors, such as epidermal growth factor (EGF), insulin growth factor, factors of the transforming growth factor-β (TGF-β) superfamily, etc. These autocrine or paracrine factors regulate follicle development through acting on gonadotropin and steroid hormones.

As a member of the TGF-β family, bone morphogenetic protein-6 (BMP-6) plays a regulatory role in various processes. BMP-6 protein expression can be detected in oocytes and granulosa cells in humans, cattle, sheep, swine and rats.[1–6] The expression of BMP-6 protein undergoes a dynamic change during development of follicle and luteinization. At present, the effect of BMP-6 on steroidogenesis is extensively studied by incubating granulosa cells and (or) theca cells *in vitro*. BMP-6 is regarded as the corpus luteum inhibitor, since it inhibits progesterone production to prevent early maturation of follicles.[3,7–9] The effects of BMP-6 on estradiol synthesis and proliferation of the granulosa cells and theca cells vary among different species.[7–11] In addition, it has been demonstrated that the reproductive capacity (litter size) of BMP-6 knockout female mice is not notably reduced.[12] However, Koji et al. [13] reported that the litter size, natural ovulation rate and development of zygote after fertilization were markedly decreased in BMP-6 knockout mice.

In this study, the BMP-6 expression in mouse follicles was detected both *in vivo* and *in vitro*. The effects of recombinant human BMP-6 (rhBMP-6) at different concentrations on the development and maturation of preantral follicles *in vitro* were also investigated.

## Materials and methods

### Animals

The female (C57b1/6×DBA/2) F1 mice were obtained from The Experimental Animal Centre of Nantong University. All animal care and procedures were approved by the Animal Care and Use Committee of Nantong University and all the experiments were conducted in accordance with institutional guidelines.

### Immunofluorescence analysis

Bilateral ovaries were obtained from 14-, 20-, 24-day-old mice. After fixation with 4% paraformaldehyde, the samples were dehydrated with sucrose at different concentrations. Cumulus oocyte complexes (COCs) and gobbets of granulosa cells were collected from 6-day and 10-day culture and were also fixed with 4% paraformaldehyde. The ovaries and gobbets of granulosa cells were then sliced into 10 μm thick sections with a cryostat microtome (Leica CM 1900) and maintained at 4 °C overnight. On the following day, the sections and COCs were washed with 0.01 mmol/L phosphate buffered saline (PBS; pH 7.0–7.2) for 10 min. After washing three times, samples were immersed in blocking buffer containing 10% goat serum (Santa Cruz Corporation, USA) for 30 min at 37 °C. Then, the sections and COCs were incubated with rabbit anti-mouse BMP-6 IgG (1:200, Santa Cruz Corporation, USA) for 24 h at 4 °C. After washing three times with 0.01 mmol/L PBS (pH 7.0–7.2) for 10 min, the samples were incubated with fluorescein isothiocyanate (FITC)–goat anti-rabbit IgG (Santa Cruz Corporation, USA) at 37 °C for 3 h. After washing with 0.01 mmol/L PBS (pH 7.0–7.2) three times, the sections and COCs were added with glycerine and were covered with a coverslip for observation under a fluorescence microscope (Leica DM 400 B, Germany).

### Western blot analysis

For protein extraction, bilateral ovaries were obtained from 20- and 24-day-old mice. Follicles were obtained from 14-day-old mice and cultured for 0, 6 and 10 days (500 follicles each) *in vitro*. Total proteins were extracted from these ovaries and follicles with radioimmunoprecipitation assay lysis buffer (plus 1% phenylmethanesulfonyl fluoride, Beyotime Corporation, China). Then, the protein extracts were heated at 95 °C for 5 min and quantified with a NanoPhotometer (IMPLEN, USA). After adding 2× sodium dodecyl sulfate polyacrylamide gel electrophoresis (SDS-PAGE) loading buffer, aliquots containing an equal amount of protein in each group were loaded onto a 10% SDS-PAGE gel (Keygen Biological Corporation, China), and run at 110 V for about 90 min. Then, the proteins in the gel were transferred onto a polyvinylidene fluoride (PVDF) membrane with a semi-dry transfer cell for 1 h. After blocking with 5% non-fat milk, the PVDF membrane transferred with proteins was incubated overnight at 4 °C with rabbit anti-mouse BMP-6 antibodies (1:100) or mouse anti-β-actin antibodies (1:1000, Beyotime Corporation, China). The PVDF membrane was then incubated with horse radish peroxidase (HRP) goat anti-rabbit antibodies or HRP–goat anti-mouse antibodies (1:1000, Beyotime Corporation, China) at room temperature for 3 h, and placed in a ChemiDOC™ XRS^+^ imaging system (BIO-RAD, USA). Photographs were taken with the Image Lab 3 software after covering with enhancing developer solution. The relative grey values were analysed with IMAGE J software (NIH, USA).

### Preantral follicles culture *in vitro*

Ovaries were obtained from 14-day-old mice and incubated with L-15 medium containing 10% fetal bovine serum (FBS), 50 U/mL of penicillin and 50 mg/mL of streptomycin. The typical preantral follicles were individually placed in 20 droplets of Alpha-Modified Eagle Medium containing 5% FBS, 1% insulin–transferrin–selenium, 0.1 mIU/mL of recombinant follicle-stimulating hormone, 50 U/mL of penicillin and 50 mg/mL of streptomycin, and were then covered with 5 mL mineral oil per 60 mm culture dish and incubated at 37 °C in a 5% CO_2_ environment. The average diameter of each follicle was measured overnight, and only follicles with a diameter of 110 μm to 160 μm were incubated for further 10 days.

The cultured preantral follicles were divided into the following five groups: control group (*n* = 20); group A, incubated with 50 ng/mL of rhBMP-6 (Sigma, USA) for 11 days (*n* = 20); group B, incubated with 50 ng/mL of rhBMP-6 for 8 days (*n* = 20); group C, incubated with 100 ng/mL of rhBMP-6 for 11 days (*n* = 20) and group D, incubated with 100 ng/mL of rhBMP-6 for eight days (*n* = 20). In all groups, rhBMP-6 was added on day 0. Follicles in groups A and C were cultured in the presence of rhBMP-6 for 11 days. For follicles in groups B and D, rhBMP-6 was removed on day 8 of culture. Then, the follicles in groups B and D were further cultured for three days without rhBMP-6.

The medium was refreshed semi-quantitatively on alternate days and the follicle development was observed and recorded. The spent medium was collected and stored at −20 °C for hormone level measurement. To stimulate follicle ovulation, the culture was refreshed with new medium containing 2.5 U/mL of human chorionic gonadotropin and 5 ng/mL of EGF on day 10. After 16 h of incubation, the surrounding granulosa cells from COCs extract were removed and the nude oocytes were observed by an inverted microscope (OLYMPUS IX70, Japan) and classified into germinal vesicle (GV), germinal vesicle breakdown (GVBD) and M II phase. The maturation rate was the ratio of M II phase oocytes to the total number of follicles.

### Enzyme-linked immunosorbent assay (ELISA)

The hormone levels of estradiol (E_2_) and progesterone (P_4_) in the spent medium from the five different groups were examined by ELISA after incubation for 6, 10 and 11 days. The E_2_ and P_4_ concentrations in the tested samples were calculated based on a standard curve obtained according to the optical density values of standard samples.

### Reverse transcription quantitative polymerase chain reaction (RT-qPCR)

Total RNA was extracted from granulosa cells and theca cells from the five different groups cultured for 6, 10 and 11 days. RNA was extracted with TRIzol® (Life Technologies, USA). The extracted RNA was transcribed into cDNA by a RevertAid First Strand cDNA Synthesis Kit (Thermo Scientific Corporation, USA) with specific primers (Table 1), following the manufacturer's instructions. Beta-actin was used as an internal control. The relative expression level of genes encoding P450scc, stAR, 3β-HSD, CYP19A1 and 17β-HSD was detected by the SYBR Green qPCR kit (Thermo Scientific Corporation, USA).

**Table 1.** Primer sequences.

Gene	Gene symbol	Primer sequence (5′–3′)
Beta-actin	Actb	F: GGCACAGTGTGGGTGAC R: CTGGCACCACACCTTCTA
Cytochrome P450 cholesterol side-chain cleavage	Cyp11a1	F: AGATCCCTTCCCCTGGCGACAATG R: CGCATGAGAAGAGTATCGACGCATC
Steroidogenic acute regulatory protein	StAR	F: CAGGGAGAGGTGGCTATGCA R: CCGTGTCTTTTCCAATCCTCTG
3b-Hydroxysteroid dehydrogenase 1	HSD3b1	F: CAGGAGAAAGAACTGCAGGAGGTC R: GCACACTTGCTTGAACACAGGC
17b-hydroxysteroid dehydrogenase 1	HSD17b1	F: ACTGTGCCAGCAAGTTTGCG R: AAGCGGTTCGTGGAGAAGTAG
Cytochrome P450 aromatase	Cyp19a1	F: CATGGTCCCGCAAACTGTGA R: GTAGTAGTTGCAGGCACTTC

### Morphology and distribution of chromosome and spindle

M II (meiosis II) oocytes collected from different groups were fixed with 4% paraformaldehyde at 4 °C for 24 h. After washing with 0.01 mol/L PBS (pH 7.0–7.2) for three times (each time for 5 min), the oocytes were incubated with PBS containing 1% Triton-X 100 at 37 °C for overnight to enhance the oocyte membrane permeability. On the second day, the oocytes were transferred to blocking buffer (PBS containing 1% BSA and 0.01% Triton-X 100) and cultured at 37 °C for 1 h after washing with 0.01 mol/L PBS (pH 7.0–7.2) three times (each time for 5 min). The cultured oocytes were mixed with anti-α-tubulin–FITC (1:100 in blocking buffer, Sigma, USA) and incubated at 37 °C for 1 h. After washing with 0.01 mol/L PBS (pH 7.0–7.2), the oocytes were mixed with 5 μg/mL of Hoechst 33342 (Sigma, USA) and incubated for 15 min at room temperature. The oocytes were finally covered with glass slides and observed under a confocal microscope (Leica CXT 588, Germany).

### Statistical analysis

All data were analysed by SPSS 17.0 software. Experiments were repeated three times. Data were expressed as mean ± standard error of the means. The differences in the growth rate and maturation rate were assessed by chi-square test. The differences in steroid hormone levels and gene expression levels were assessed by one-way ANOVA. Statistical significance was assumed when *P* < 0.05.

## Results and discussion

### Location of BMP-6 protein in follicles *in vivo* and * in vitro*

**Figure 1.**
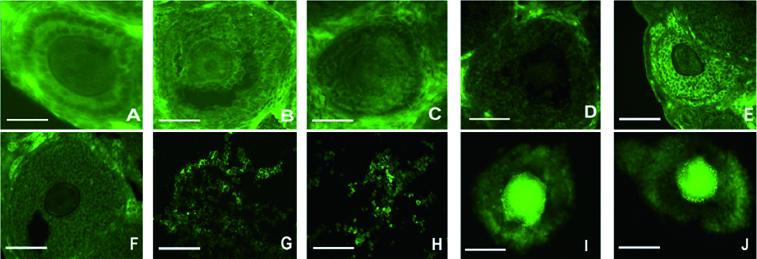
*In vivo* and *in vitro* observation of BMP-6 expression in follicles. BMP-6 expression was observed by immunofluorescence. *In vivo* expression of BMP-6 in primary follicles on day 14 (A), day 20 (B) and day 24 (C). *In vivo* expression of BMP-6 in the secondary follicles on day 14 (D), day 20 (E) and day 24 (F). *In vitro* expression of BMP-6 in granulosa cells from follicles on day 6 (G) and day 10 (H). *In vitro* expression of BMP-6 in the COC from follicles on day 6 (I) and day 10 (J). Bar = 100 μm.

To determine the location of BMP-6 protein in follicles *in vivo* and *in vitro*, its immunofluorescence was microscopically observed in ovaries *in vivo* and in follicles from culture. As shown in Figure 1, BMP-6 protein was expressed *in vivo* in the primary follicles on day 14 (Figure 1(A)), day 20 (Figure 1(B)) and day 24 (Figure 1 (C)), as well as in the secondary follicles on day 14 (Figure 1(D)), day 20 (Figure 1(E)) and day 24 (Figure 1(F)). BMP-6 protein was also expressed *in vitro* in granulosa cells from cultured follicles on day 6 (Figure 1(G)) and day 10 (Figure 1(H)) and in the COC from follicles on day 6 (Figure 1(I)) and day 10 (Figure 1(J)). The immunofluorescence results demonstrated that BMP-6 protein was expressed in the primary and secondary follicles *in vivo*, as well as in follicles cultured for 6 and 10 days *in vitro*.

### Quantitative expression of BMP-6 protein in follicles *in vivo* and *in vitro*

**Figure 2.**
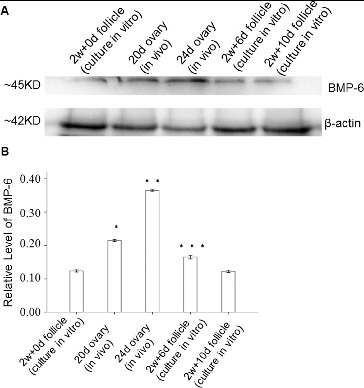
Expression levels of BMP-6 in ovaries *in vivo* and in follicles cultured *in vitro*. Follicles were cultured for 0, 6 and 10 days (500 each) *in vitro*. Representative western blot results (A); quantitative western blot results (B). β-actin was used as an internal control. *P < 0.05, compared with the control group.

To determine the expression levels of BMP-6 protein in follicles *in vivo* and *in vitro*, western blot was performed. The western blot results showed that the BMP-6 expression level in follicles *in vivo* increased with time. *In vitro*, when compared with the preantral follicles from the initial culture (day 0), there was an increase in the BMP-6 expression level on day 6. However, the BMP-6 expression level declined in the antral follicles from the 10-day-old culture as compared with the control (Figure 2).

### Effect of rhBMP-6 on follicle morphology during the development of preantral follicles *in vitro*

**Figure 3.**
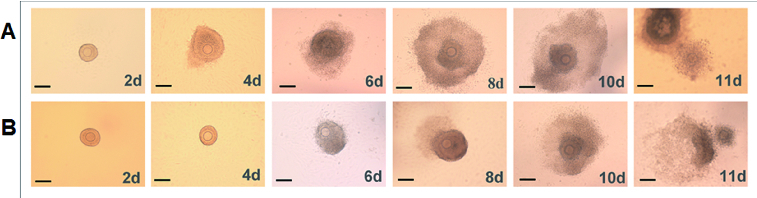
Effect of rhBMP-6 on the morphology of preantral follicles *in vitro*. Follicles under normal development (A) or abnormal development (B), on day 2, 4, 6, 8, 10 and 11. Bar = 100 μm.

To determine the effect of rhBMP-6 on the development of preantral follicles *in vitro*, preantral follicles were isolated and cultured *in vitro* in the presence of rhBMP-6. The follicle morphology in different groups containing 0, 50 and 100 ng/mL rhBMP-6 was observed. Similar to control follicles without rhBMP-6 treatment, there were both normally and abnormally developed follicles after rhBMP-6 treatment. Thus, the follicle morphology during development was not obviously affected by rhBMP-6. Figure 3 shows representative morphologies of normal (Figure 3(A)) and abnormal follicles (Figure 3(B)). As shown in Figure 3(A), the normal follicles were attached to the bottom of the Petri dish on day 2, which was followed by granulosa cells breaking through the basement membrane and proliferation on day 4. The proliferation of granulosa cells continued and bulged toward the vicinity of the follicles on day 6. Finally, the follicular antrum appeared on day 8 and became enlarged on day 10 (Figure 3(A)). On the contrary, the above-described morphologies were all abolished in abnormally developed follicles (Figure 3(B)). The abnormal follicles were not attached to the bottom of the Petri dish on day 2 and, on day 4, granular cells did not proliferate and the basement membrane was unbroken. The granular cells began to proliferate on day 6. In addition, the follicular antrum appeared on day 10 and not on day 8. Nevertheless, the COCs were ejected after ovulation induction.

### Effect of rhBMP-6 on the maturation of preantral follicles *in vitro*

**Figure 4.**
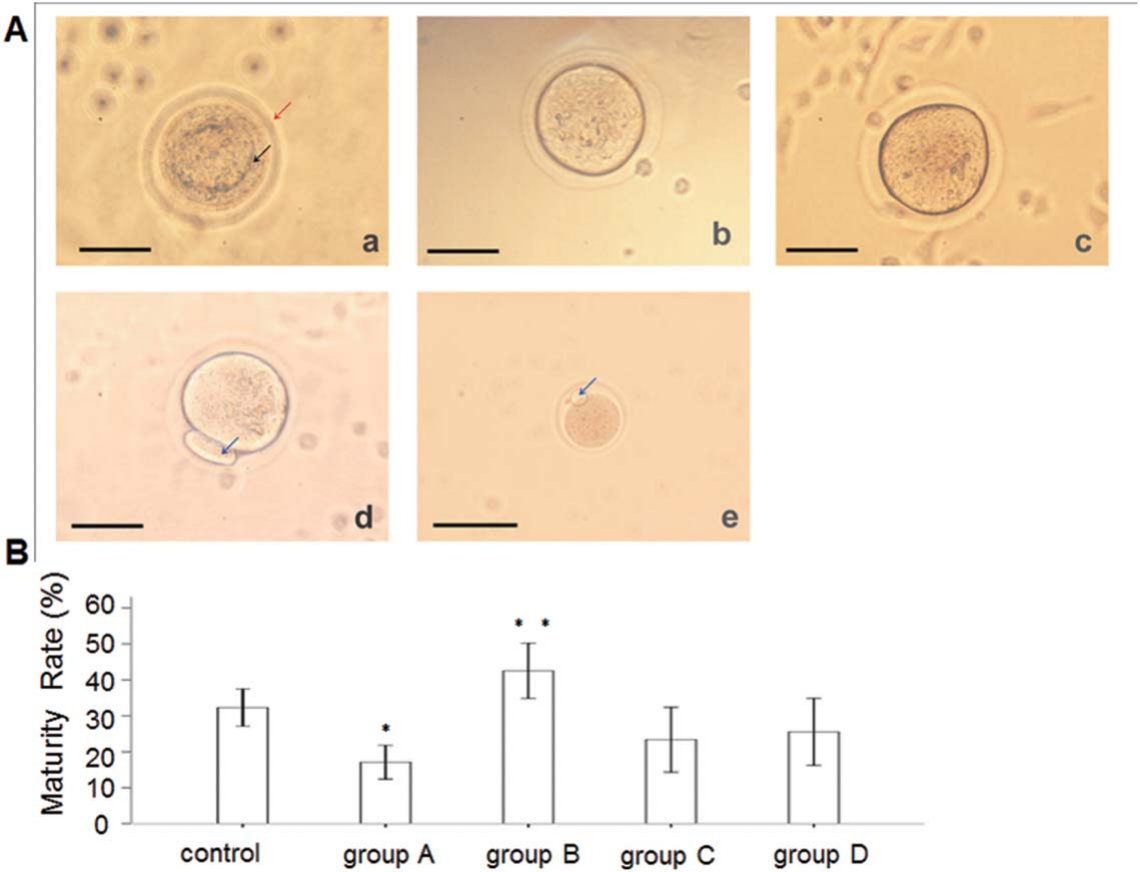
Effect of rhBMP-6 on the maturation rate of preantral follicles *in vitro*. (A) *In vitro* observation under inverted microscope (bar = 50 μm): GV oocyte (a), GVBD oocyte (b), M I oocyte (c), M II oocyte (d) and *in vivo* observation of M II oocyte (e); GV (the upper arrow in (a)), zona pellucida (the lower arrow in (a)) and first polar body (the arrows in (d) and (e)). (B) Maturation rates of follicles in different groups: control, group A (incubation with 50 ng/mL rhBMP-6 for 11 days), group B (incubation with 50 ng/mL rhBMP-6 for 8 days), group C (incubation with 100 ng/mL rhBMP-6 for 11 days) and group D (incubation with 100 ng/mL rhBMP-6 for 8 days). *P < 0.05, compared with the control group.

To observe the effect of rhBMP-6 on the maturation of preantral follicles *in vitro*, preantral follicles were cultured *in vitro* in the presence of rhBMP-6 and, after ovulatory stimulation, follicle maturation was observed. Figure 4(A) illustrates the maturation phases of follicles, including GV phase (a), GVBD phase (b), M I phase (c) and M II phase (d). Oocytes in GVBD phase have the capacity for completing the meiosis. Oocytes in M I phase are immature, while the ones in M II phase are mature. An M II oocyte (Figure 4[A(e)]) observed *in vivo* is also shown. Then, we analysed the maturation rate in different groups by calculating the ratio of M II phase oocytes to the total number of follicles. Compared with the control group, the oocyte maturation rate in group B (incubated with 50 ng/mL BMP-6 for eight days) was significantly increased (*P* < 0.05). However, the maturation of follicles from group A (incubated with 50 ng/mL BMP-6 for 11 days) was remarkably inhibited (*P* < 0.05). There was no obvious difference observed in either group C or group D (incubated with 100 ng/mL BMP-6 for 11 and 8 days, respectively) (Figure 4(B)).

### Chromosome distribution and meiotic spindle morphology

**Figure 5.**
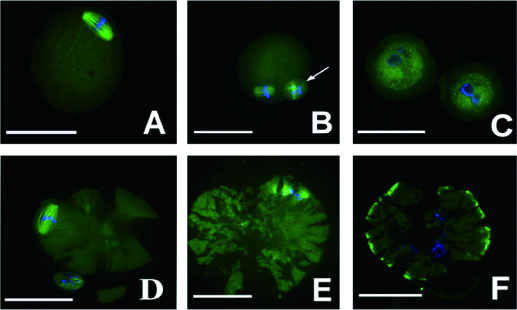
Morphology and distribution of chromosomes and spindle. Images were observed under confocal microscope. Chromosome and spindle in normal M II oocytes (A and B); M II oocytes whose size was the same with that of the first polar body in group D (C); cataclastic M II oocytes with normal chromosome and spindle in group A (D); cataclastic M II oocytes with the chromosomes decondensed and the spindle fibres disintegrated in group A (E and F). First polar body (white arrow); groups are described in the text to Figure 4. Bar = 100 μm.

To further determine the maturation of the nucleus in M II oocytes, the chromosome distribution and meiotic spindle morphology was observed in an immunofluorescence assay. The immunofluorescence results in Figure 5(A) and 5(B) show the chromosome distribution and meiotic spindle morphology in normal M II oocytes. We observed that there were 12 oocytes with a cracked cell membrane in group A, in which the chromosomes became decondensed and the spindle fibres disintegrated in nine cataclastic ones (Figure 5(E) and 5(F)), and no abnormalities in the chromosomes and the spindle were present in the normal M II oocytes (Figure 5(D)). In group D, only one M II oocyte was observed, showing approximately the same size as the first polar body; there was annular distribution of chromosomes without the presence of a spindle (Figure 5(C)). There were no abnormalities in the chromosomes and the spindle in the other groups.

### Levels of E_2_ and P_4_

**Figure 6.**
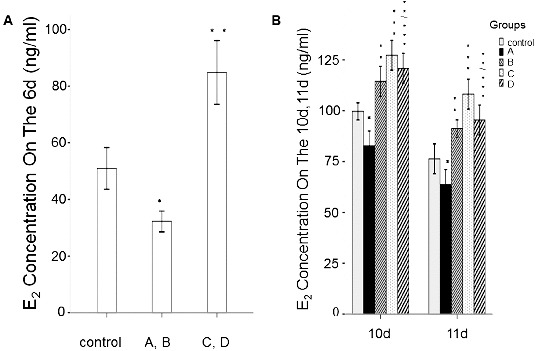
Expression levels of E_2_ in different groups in spent medium *in vitro*. ELISA results. Concentrations of E_2_ in spent medium on day 6 (A) and on day 10 and day 11 (B). Groups are described in the text to Figure 4. *P < 0.05, compared with the control group.

**Figure 7.**
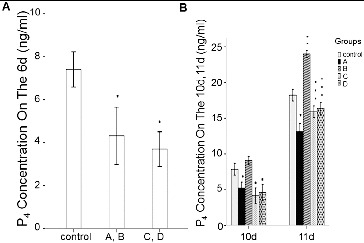
Expression levels of P_4_ in different groups in spent medium *in vitro*. ELISA results. Concentrations of P_4_ in spent medium on day 6 (A) and on day 10 and day 11 (B). Groups are described in the text to Figure 4. *P < 0.05, compared with the control group.

To determine the effect of rhBMP-6 on steroid hormones, the levels of E_2_ and P_4_ were detected by ELISA. After culturing the follicles for 6, 10 and 11 days, the spent medium from each group was collected for steroid hormone analysis. As shown in Figures 6 and 7, the E_2_ and P_4_ concentrations in group A and group B were all significantly lower than those in the control group (*P* < 0.05). However, the E_2_ and P_4_ levels were increased in group B on day 10 and day 11. In groups C and D, the addition of rhBMP-6 resulted in higher E_2_ concentration (Figure 6) (*P* < 0.05) and lower P_4_ level (Figure 7) (*P* < 0.05).

### Expression of genes encoding enzymes from the steroid hormone synthesis pathway

**Figure 8.**
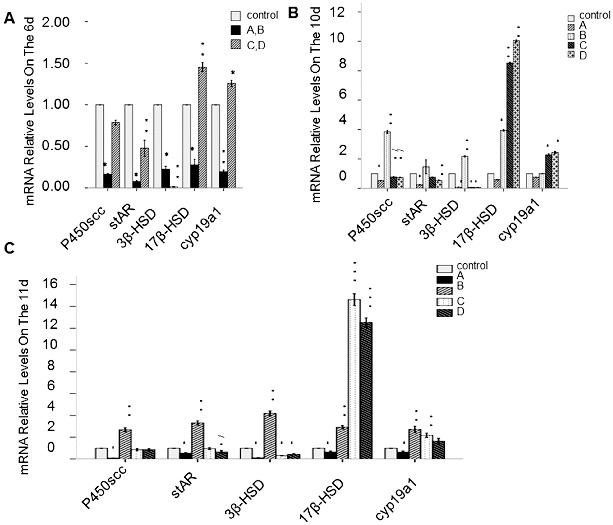
Expression of genes encoding selected regulatory enzymes involved in steroid hormone synthesis. RT-qPCR results. Expression of P450scc, stAR, 3β-HSD, 17β-HSD and cyp19a1 at the mRNA level on day 6 (A), day 10 (B) and day 11 (C). Groups are described in the text to Figure 4. Data from 20 individual follicles in three independent experiments. *P < 0.05, compared with the control group.

To determine the effect of rhBMP-6 on genes encoding enzymes with key roles in steroid hormone synthesis, RT-qPCR was performed. The expression of the following genes was analysed: P450scc, stAR, 3β-HSD, 17β-HSD and cyp19a1. In group A, the relative expression levels of the studied genes at the mRNA level in granular and theca cells were significantly decreased with culture time (Figure 8) (*P* < 0.05). However, in group B, the expression levels of these genes were significantly increased (*P* < 0.05) on day 10 and day 11 (with the exception of stAR levels on day 10). In groups C and D, the level of the 3β-HSD mRNA was significantly lower (*P* < 0.05), but the levels of the 17β-HSD and cyp19a1 mRNAs were remarkably higher (*P* < 0.05). Compared with the control, no significant difference in the P450scc mRNA level or the stAR mRNA level was observed (Figure 8).

### Comparative analysis

*In vitro* culture of preantral follicles is not only important in clinical-assisted reproduction, but is also indispensable in basic research on the mechanisms of follicles development and maturation and the factors that affect these processes. Although *in vitro* culture technology has been applied for preantral follicles from many animals, the maturation rate of oocytes was shown to be unsatisfactory, with approximately more than 50% of mature oocytes (M II oocytes) failing to fertilize or develop into an early embryo.[14]

Since BMP-6 is a member of the TGF-β superfamily, it plays an important part in all body systems. The expression of BMP-6 in ovaries varies among different species. Previous studies have demonstrated that BMP-6 mRNA is expressed in oocytes and granular cells of humans, cattle, sheep, swine and rats,[1–6] but fewer studies have been conducted with mice. In this study, BMP-6 was detected in mouse follicles both *in vivo* and *in vitro*, and its level in the former level was higher than that in the latter. Thus, we hypothesize that BMP-6 may participate in the development and maturation of follicles. In addition, our observation that the expression level of BMP-6 was increased on day 6 but, on day 10, declined to the same level as that on day 0, is in agreement with previous reports showing dynamic expression changes for BMP-6 during follicles development.[3,15]

In this research, cells were incubated with rhBMP-6 *in vitro* for different times and comparisons were conducted through different maturation stages. Our result that exogenous BMP-6 had no effect on morphological features during *in vitro* preantral follicles development is in accordance with previous reports for granular cells and theca cells from rats.[5,8] However, Brankin et al. [10] reported that BMP-6 accelerated the proliferation of porcine granular and theca cells *in vitro*. Isana et al. [16] also found that BMP-6 augmented the diameter of the secondary follicles in sheep and promoted the follicular antrum formation *in vitro*. These contradictory reports suggest that the effect of BMP-6 on granular and theca cells proliferation differs from species to species.

Although, in our study, exogenous BMP-6 did not appear to affect the morphology of preantral follicles *in vitro*, their maturation was accelerated after eight days with 50 ng/mL BMP-6 and declined after 11 days of incubation with the same concentration of BMP-6. Moreover, there were no significant differences between the maturation rate following addition of 100 ng/mL BMP-6 and that in the control. Since the developmental capacity of oocytes from M I to M II can be affected by many factors, including steroid hormone levels (e.g., E_2_ and P_4_), downstream signalling pathways and cytokines, BMP-6 may be involved in a relatively complex mechanism.

The result from our RT-qPCR assay showed that BMP-6 at a concentration of 50 ng/mL lowered the mRNA levels of the five studied genes encoding enzymes involved in steroid synthesis. After removal of BMP-6 on day 8, the inhibition effect was weakened, and the mRNA levels were returned to normal level. BMP-6 at a higher concentration (100 ng/mL) suppressed the transcription of the gene for 3β-HSD, which takes part in P_4_ synthesis regulation, and enhanced the expression (on mRNA level) of 17β-HSD and CYP19A1, which are associated with E_2_ synthesis. These mRNA expression levels were coincident with the alteration of E_2_ and P_4_ concentration in the spent culture medium.

The results from this study strongly suggest that the decrease in P_4_ caused by 50 ng/mL BMP-6 is beneficial for the prevention of follicles to luteinize too early and for accumulation of RNA, proteins and energy in oocytes. After removal of BMP-6, the contents of E_2_ and P_4_ in the culture medium increased significantly, resulting in a remarkable improvement in the maturation rate, while low E_2_ and P_4_ concentrations led to a decrease in the maturation rate. These results prove the vital role of E_2_ and P_4_ in the maturation of follicles.

The increase in E_2_ caused by 100 ng/mL BMP-6, however, is inconsistent with previous results obtained in rats.[7,8] High concentrations of E_2_ may partly inhibit P_4_ synthesis, or likely activate other factors stimulating follicles to mature simultaneously, which could have led to no significant changes in the maturation rate of oocytes *in vitro*.

The chromosome distribution and meiotic spindle morphology in M II oocytes are important for fertilization and the development of embryo. In line with this, our results showed that, after 11 days of incubation with 50 ng/mL BMP-6, the chromosomes and spindles in most of the fragmented M II oocytes were also disorganized. Abnormal chromosome and spindle were absent in normal M II oocytes.

In summary, BMP-6 was shown to be able to promote the maturation of preantral follicles *in vitro* in a concentration- and time-dependent manner and may affect the maturation of oocytes through regulating steroid hormone synthesis.

## Conclusions

In this study, the stimulatory role of BMP-6 on the maturation rate of mouse oocytes *in vitro* provide further evidence that the expression of BMP-6 in follicles undergoes dynamic changes during follicle development and BMP-6 is involved in the growth and maturation of oocytes. BMP-6 can influence the expression of steroidogenesis-related genes to further affect the steroid hormone synthesis and/or secretion. To better understand the precise role of BMP-6 and its mechanism of action, further investigations are needed.
